# Prototyping Crop Traits Retrieval Models for CHIME: Dimensionality Reduction Strategies Applied to PRISMA Data

**DOI:** 10.3390/rs14102448

**Published:** 2022-05-19

**Authors:** Ana B. Pascual-Venteo, Enrique Portalés, Katja Berger, Giulia Tagliabue, Jose L. Garcia, Adrián Pérez-Suay, Juan Pablo Rivera-Caicedo, Jochem Verrelst

**Affiliations:** 1 Image Processing Laboratory (IPL), University of Valencia, C/Catedrático José Beltran 2, 46980 Paterna, Valencia, Spain; 2 Department of Geography, Ludwig-Maximilians-Universität München (LMU), Luisenstr. 37, 80333 Munich, Germany; 3 Remote Sensing of Environmental Dynamics Laboratory (LTDA), University of Milano—Bicocca, Piazza della Scienza 1, 20126 Milano, Italy; 4 Secretary of Research and Graduate Studies, Consejo Nacional de Ciencia y Tecnología, Universidad Autónoma de Nayarit, Tepic 63155, Nayarit, Mexico

**Keywords:** PRISMA, CHIME, hybrid methods, biochemical and biophysical traits, Gaussian process regression, active learning, principal component analysis, feature selection

## Abstract

In preparation for new-generation imaging spectrometer missions and the accompanying unprecedented inflow of hyperspectral data, optimized models are needed to generate vegetation traits routinely. Hybrid models, combining radiative transfer models with machine learning algorithms, are preferred, however, dealing with spectral collinearity imposes an additional challenge. In this study, we analyzed two spectral dimensionality reduction methods: principal component analysis (PCA) and band ranking (BR), embedded in a hybrid workflow for the retrieval of specific leaf area (SLA), leaf area index (LAI), canopy water content (CWC), canopy chlorophyll content (CCC), the fraction of absorbed photosynthetic active radiation (FAPAR), and fractional vegetation cover (FVC). The SCOPE model was used to simulate training data sets, which were optimized with active learning. Gaussian process regression (GPR) algorithms were trained over the simulations to obtain trait-specific models. The inclusion of PCA and BR with 20 features led to the so-called GPR-20PCA and GPR-20BR models. The 20PCA models encompassed over 99.95% cumulative variance of the full spectral data, while the GPR-20BR models were based on the 20 most sensitive bands. Validation against in situ data obtained moderate to optimal results with normalized root mean squared error (NRMSE) from 13.9% (CWC) to 22.3% (CCC) for GPR-20PCA models, and NRMSE from 19.6% (CWC) to 29.1% (SLA) for GPR-20BR models. Overall, the GPR-20PCA slightly outperformed the GPR-20BR models for all six variables. To demonstrate mapping capabilities, both models were tested on a PRecursore IperSpettrale della Missione Applicativa (PRISMA) scene, spectrally resampled to Copernicus Hyperspectral Imaging Mission for the Environment (CHIME), over an agricultural test site (Jolanda di Savoia, Italy). The two strategies obtained plausible spatial patterns, and consistency between the two models was highest for FVC and LAI (*R*^2^ = 0.91, *R*^2^ = 0.86) and lowest for SLA mapping (*R*^2^ = 0.53). From these findings, we recommend implementing GPR-20PCA models as the most efficient strategy for the retrieval of multiple crop traits from hyperspectral data streams. Hence, this workflow will support and facilitate the preparations of traits retrieval models from the next-generation operational CHIME.

## 1 Introduction

As the world population is expected to continue to grow in the next decades, food security will become a crucial problem requiring political decisions and strategic solutions [1]. Optical remote sensing technologies have been employed to monitor the terrestrial Earth surface routinely and thus provide a viable tool to measure fundamental crop traits in the context of sustainable agriculture [2]. Among a diversity of platforms, satellite sensors can acquire data over vast cultivated regions, which allows the generation of efficient and useful products for managing agricultural systems. In the next coming years, an increasing number of spaceborne imaging spectroscopy missions will complement current multispectral Earth observation (EO) systems, such as the Copernicus Sentinel-2 from the European Space Agency (ESA), leading to an unprecedented flow of data in high spectral dimensionality [3]. These hyperspectral missions include, among others, the PRecursore IperSpettrale della Missione Applicativa (PRISMA) [4], launched on 22 March 2019, and the Environmental Mapping and Analysis Program (EnMAP) [5], launched on 1 April 2022. Following the two precursor missions, forthcoming operational missions are planned, such as the FLuorescence EXplorer (FLEX) [6], the NASA Surface Biology and Geology observing system (SBG) [7], and the Copernicus Hyperspectral Imaging Mission for the Environment (CHIME) [8].

CHIME will be designed to provide routine hyperspectral observations through the Copernicus Programme starting between 2025 and 2030 [3], thus complementing the Sentinel-2 multispectral mission [9]. The CHIME sensor is built upon a pushbroom concept providing contiguous spectra assembled by more than 200 narrow bands in the 400−2500 nm spectral range. The spectral sampling interval will be < 10 nm and each sensor will record at a spatial sampling distance of 30 m. The mission will provide data in a repeat cycle of 20 to 25 days for a single satellite and 10−12.5 days for two satellites using a sun-synchronous orbit [10].

CHIME’s main objective will be to improve and develop new services focusing on the precise management of natural resources to support a range of related policies and decisions. Within the natural resources management, a primary pillar will be ‘sustainable agriculture and food security,’ including, among others, food nutrition and quality [11]. To support this, CHIME shall deliver required quantitative measures of essential crop traits in space and time and high accuracy meeting user requirements within the agricultural services [11]. In this way, the mission will support European Union (EU)-related policies, such as the green and performance-based EU Common Agricultural Policy (see: https://ec.europa.eu/info/food-farming-fisheries/key-policies/common-agricultural-policy, accessed on 15 March 2022), aiming for sustainable agricultural management. With the background of these green EU goals, dedicated retrieval algorithms that can be easily implemented into operational schemes to obtain prioritized variables need to be identified. To support the preparatory activities of CHIME, an end-to-end (E2E) instrument simulator was established, which can approximate the complete chain starting from data recording, sensor calibration, and data pre-processing to sensor products up to final surface properties mapping [12]. Following the atmospheric and geometric correction processes providing Level-2A (L2A) products, multiple vegetation traits models will be implemented in the Level-2B (L2B) module of the E2E simulator [13]. These traits encompass biochemical and biophysical vegetation properties, such as leaf and canopy chlorophyll content (*C_ab_*, CCC), specific leaf area (SLA), leaf area index (LAI), leaf and canopy water content (LWC, CWC), the fraction of absorbed photosynthetic active radiation (FAPAR), and fractional vegetation cover (FVC). Retrieval of these traits is relevant for diverse agricultural applications to support sustainable management and thus food production [14,15]. While the majority of these traits have been derived numerous times experimentally or in operational missions (see reviews Verrelst et al. [16,17]), currently no mission in space routinely provides a catalog of these vegetation products.

When it comes to the routine production of biochemical and biophysical traits from EO data, efficient retrieval methods need to be implemented. The key challenge lies in finding the trade-off between site-specific accuracy and operational continuity. An overview and elaborated taxonomy of variable retrieval methods from Earth observation data is provided by Verrelst et al. [16,17]. From the main families of retrieval methods, i.e., (1) parametric regressions, (2) nonparametric regressions, (3) physically-based methods, and (4) hybrid approaches, the last method evolved as the most appealing in operational contexts [18–26]. Hybrid strategies blend the physics described by radiative transfer models (RTM) and use the efficiency of machine learning regression algorithms (MLRAs) in a synergistic way to infer the traits of interest. Within such workflows, synthetic training data sets are firstly generated from RTM simulations describing multiple states of vegetation characteristics. Subsequently, a selected machine learning algorithm learns the nonlinear relationships between the pairs of simulated reflectance and vegetation traits to build a predictive model [16,27]. However, when hybrid methods are applied to hyperspectral data, some challenges must be overcome. Imaging spectrometers, such as CHIME, are characterized by numerous contiguous spectral bands providing a vast amount of detailed information but also contain spectral redundancy and noise [28]. Consequently, ingesting all these bands directly into an MLRA would lead to long training times and suboptimal mapping performances [27,29].

To circumvent this redundant information and improve model efficiency, dimensionality reduction (DR) in both the sampling (i) and spectral (ii) domains need to be accomplished [27]. With respect to (i), active learning (AL) methods were proposed to reduce training sample sizes and thus also final models effectively [30–32]. Traditionally used for classification [33], recently, AL techniques have been pursued to solve numerous regression problems in the context of EO data analysis targeting vegetation properties retrieval [34]. When applying AL, a machine learning algorithm can reach superior accuracies as it learns from an optimized and representative training data set [35]. In addition, computational runtime is reduced, allowing the implementation of MLRAs that require a relatively small number of training points, such as Gaussian process regression (GPR) algorithms [36,37]. GPRs are outstanding in delivering competitive performances [38] and can provide associated uncertainty looking at predictive variance estimates [39]. Consequently, they may be the preferred methods in the framework of hybrid retrieval strategies [17].

Regarding spectral dimensionality reduction (ii), we can broadly distinguish between (1) feature extraction or band selection [20,31] and (2) feature transformation, also known as feature engineering [40]. Both reduction techniques convert the spectral data into a lower-dimensional feature space, assuring that the majority of the spectral information is kept. In the case of feature extraction, a subset of the most relevant bands is selected to construct a model. Hereby we differentiate between three different methods: filter, wrapper, and embedded modeling [41,42]. In view of filter methods, traditionally, vegetation indices have been employed, extracting two or three bands and building linear relationships with the variables of interest [43–47]. However, despite straightforward implementation and successful usage in multiple studies, these methods may fail to find the correct subset of bands (or features). In addition, available (hyperspectral) information is underexploited and noise sensitivity can be enhanced if narrow bands with relatively low signal-to-noise ratios were combined [48]. For these reasons, embedded or wrapper methods should be preferred, as demonstrated by a few variable retrieval studies [2,31,49]. Feature engineering is usually based on mathematical projections, which attempt to transform the original features into an appropriate feature space. After transformation, the original meaning of the features is usually lost [40]. The most prominent method is principal component analysis (PCA) [50]. For further explanation and discussion about these methods, we refer to Berger et al. [20]. In prior studies, spectral dimensionality reduction was incorporated in hybrid strategies, either using band selection [20], but mainly using feature engineering in the form of PCA [12,13,22,23,32,51,52]. However, a direct comparison is lacking and the most efficient strategy for retrieving multiple vegetation traits from hyperspectral data sets remains to be investigated.

Altogether, with the ambition to support the upcoming CHIME with efficient retrieval methods, the overarching objective of this study was to identify the optimal hybrid strategy for deriving essential crop traits, such as SLA, LAI, CCC, CWC, FAPAR, and FVC from imaging spectroscopy data. To achieve this objective, we applied AL in the sampling domain to obtain representative training samples and compared two different spectral feature reduction strategies. Direct and indirect evaluation of the retrieval models is provided by exploring a field data set. As CHIME is yet to be launched, in anticipation of the upcoming hyperspectral data stream, the developed models will be applied and tested on a hyperspectral PRISMA image covering large cultivated areas.

## 2 Material & Methods

### 2.1 Study Design & Workflow

**Figure 1:**
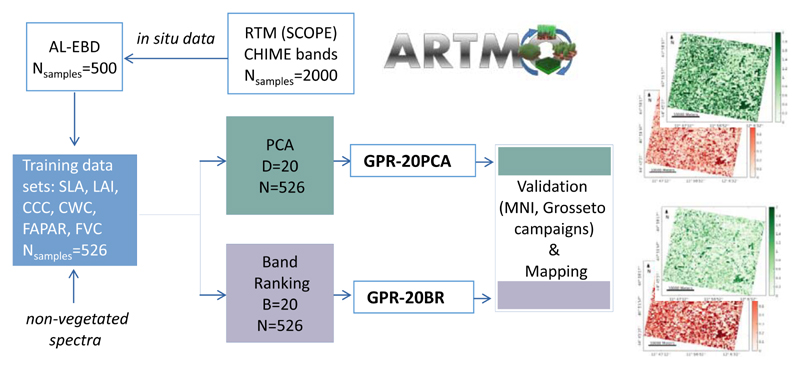
Workflow of the two pursued hybrid retrieval strategies for crop traits mapping. N: number of training samples (full pool, AL optimized), D: number of components, B: number of bands used for training.

The foundations of this study are based on a hybrid method, combining RTMs with machine learning algorithms, and applying dimensionality reduction in the sampling and in the spectral domains. Figure 1 delineates the workflow with the two pursued retrieval strategies consisting of six main steps, which will be detailed in the following subsections.

1. Generating a training database with an RTM (see Section 2.2);
2. Applying AL methods to reduce and optimize the training data sets for each variable (see Section 2.3);
3. Training and validation using GPR (Section 2.4);
4. Reducing dimensionality of simulated and measured spectra with: (i) PCA and (ii) an iterative band ranking (BR) procedure (see Section 2.5);
5. Mapping using PRISMA scenes, resampled to CHIME, over cultivated areas of the agricultural site close to Jolanda di Savoia, Italy (data set description see Section 2.6).

For all analyses performed in our study, the scientific Automated Radiative Transfer Models Operator (ARTMO, https://artmotoolbox.com/, accessed on 2 January 2022 [53]) software framework was employed. ARTMO includes the machine learning regression algorithm (MLRA) toolbox with an integrated active learning module [32] for retrieval applications. Different kinds of MLRAs, AL methods, and spectral dimensionality reduction (PCA, GPR-BAT tool) as well as sampling strategies using RTMs can be tested and applied.

### 2.2 Training Database Establishment

Ideally, a training data set for an ML algorithm should mimic the spectra encountered in real scenes as realistically as possible. This can be achieved by generating multiple combinations of vegetation variables with the RTM and applying wide statistical distributions.

We selected the Soil Canopy Observation, Photochemistry and Energy fluxes (SCOPE) model (version 1.7) [54] for our purpose. SCOPE is based on a modular architecture, encoding knowledge of radiative transfer, micrometeorology, and plant physiology. The different modules can be used separately or integrated into a cascade, exchanging inputs and outputs. Within SCOPE, optical properties of the leaves are modeled by PROSPECT-5 [55] and Fluspect [56], whereas the canopy structural properties are described by SAIL. We also chose SCOPE due to the energy balance module, which iteratively calculates heat and radiation fluxes. Therefore, it allowed for the indirect definition of FAPAR and FVC.

For the establishment of the training database, we set the ranges of the target variables (see Table 1) according to OPTICLEAF database (OPTICLEAF; http://opticleaf.ipgp.fr/, accessed on 23 December 2021), as well as similar studies using PROSPECT-4SAIL or SCOPE [16,23,27,57–60]. Leaf optical properties such as leaf chlorophyll content (*C_ab_*), leaf water content (*C_w_*), and leaf dry matter content (*C_m_*) were generated with truncated Gaussian distributions as this corresponds to their natural distributions. With respect to total leaf carotenoid content (*C_xc_*), the variable was distributed in its habitual range of variation to render spectra more realistic for the photosynthetically active radiation (PAR) region. However, the chosen distribution was uniform since this is not a target variable. Lastly, anthocyanin content (*C_ant_*) and senescent material (*C_s_*) have been set to 0 as this retrieval scheme is aimed at modeling green canopies. For the retrieval of brown (or senescent) canopies, specific retrieval strategies relying on the modeling of senescent leaf compounds [61] need to be developed. Soil reflectance was described by the Brightness-Shape-Moisture (BSM) soil reflectance model [62,63]. For all input parameters of BSM, i.e., soil moisture content (SMC), soil brightness, longitude, and latitude, the distributions were set to Gaussian. Although these variables are not of interest for the retrieval scheme, it is necessary to account for their variability in the training data to make the spectra as realistic as possible. Illumination and viewing variables, i.e., sun zenith angle (SZA), observer zenith angle (OZA), and relative azimuth between sun and observer (RAA), were varied to cover the range of possible sun-sensor-target configurations for the imagery. These have uniform distributions since there is no preferred observation direction. Lastly, regarding canopy structure variables, LAI and LIDFa/b are input from the SAIL model. Though LAI is not a priority variable, it is required for the upscaling of leaf variables to canopy level (see Table 2). In this case, uniform distributions within the usual range of variation have been specified.

Given the provided ranges in Table 1, the number of randomly selected simulations resulting from the combination of the parameters was set to 2000. In other studies, [59,60] the number of performed simulations was substantially higher (e.g., order of 100,000). However, previous studies have also proven that for hybrid retrieval strategies, competitive results can be achieved with fewer but intelligently selected samples [32,34,64]. Thus, the 2000 samples generated in this training data set were subsequently used as input to a specific active learning method for selecting the most relevant samples (see Section 2.3).

Lastly, the generation of the training database required an additional step to obtain the variables selected for retrieval (see Table 2). This included upscaling of the leaf variables to the canopy level, i.e., CCC and CWC, by multiplying the corresponding leaf variables with LAI (all in g/m^2^). *C_m_* was converted into SLA by calculating its inverse. Note that the use of SCOPE allowed us to indirectly define FVC and FAPAR, which rely on the primary variables LAI and *C_ab_*.

FAPAR was calculated as the ratio between the downward direct and diffuse photo-synthetically active radiation (PAR, 400−700 nm) and upward fluxes of PAR, as calculated in SCOPE [54]. FVC is obtained empirically from the gap fraction (*P*) at nadir, by the expression defined in De Grave et al. [23] as follows in Equation (1):

(1) P=exp(−kxLAI)

where *k* is the extinction coefficient. Given this relation, we can obtain FVC in Equation (2) as:

(2) FVC=1−P

Though these variables were not defined as a priority for CHIME, they are essential to disentangle structural and biochemical influences on the reflected spectral signals.

### 2.3 Sample Reduction: Active Learning

AL aims to optimize training datasets through intelligent sampling using an iterative procedure. In the context of regression for terrestrial EO data analysis, AL techniques are typically categorized into two groups: *uncertainty* and *diversity* [64]. In a recent survey [34] it was observed that choosing samples according to their diversity often led to optimal results. Particularly, the Euclidean distance-based diversity (EBD) method was the best performing in most reviewed studies, and, therefore, we chose to adapt this method for our study. The EBD method [65] selects those samples out of the pool that are distant from the already included ones in the training set, using squared Euclidean distance (Equation (3)):

(3) $$d_{E}={\left\|x_{u}-x_{l}\right\|}_{2}^{2},$$

where *x_u_* is a sample from the candidate set, and *x_l_* is a sample from the training set. All distances between samples are computed and then the most remote are selected. An additional optimization option was introduced by Verrelst et al. [32]. Thereby, the AL algorithm is run against in situ data. In this way, the training database becomes optimized against real data. It must be remarked that the spectral data were compressed into principal components for running the AL procedure as GPR models require exhaustive processing times with hundreds of spectral bands. Yet, that step is only for efficient GPR running; the AL-reduced database preserves all bands. The stopping criterion was set to 500 samples to provide the optimal compromise between final model sizes and accuracy. The selection was performed using the root mean squared error (RMSE), but results will also be demonstrated with the coefficient of determination (*R*^2^) and normalized RMSE (NRMSE) in %, being RMSE divided by the range of observations.

Subsequently to the AL optimization, we added 26 non-vegetated spectra to each variable-specific training database defining respective variable values to zero. These spectra were selected from the PRISMA scene (see Section 2.7) and included bare soils, water bodies, and man-made surfaces. This step allowed one to reduce the mapping errors by augmenting the model’s ability to recognize multiple non-vegetated spectral surfaces in the scene.

### 2.4 Gaussian Process Regression

Gaussian process regression [36] algorithms have been chosen as core algorithms in the hybrid retrieval scheme as they have proven good performance in variable retrieval studies [38,66,67]. In particular, GPR models address the key question of providing uncertainties for the estimates in remote sensing products. See [16,17,37] for a rationale for using GPR as opposed to alternative statistical methods.

Notationally, the GPR model establishes a relation between the input (*B*-bands spectra) $x\in {\mathbb{R}}^{B}$ and the output variable (canopy parameter to be retrieved) y∈ℝ of the form (Equation (4)):

(4) $$\hat{y}=f\left(x\right)=\sum_{i=1}^{N}\alpha_{i}K\left(x_{i},x_{j}\right),$$

where ${\left\{x_{i}\right\}}_{i=1}^{N}$ are the spectra used in the training phase, $\alpha_{i}\in \mathbb{R}$ is the weight assigned to each one of them, and *K* is a function evaluating the similarity between the test spectrum **x** and all *N* training spectra, $x_{i}={\left[x_{i}^{1},x_{i}^{2},\ldots ,x_{i}^{B}\right]}^{⊺},\;i=1,\ldots ,N$. We used ARD Rational Quadratric Kernel:

(5) $$K\left(x_{i},x_{j}|\theta \right)=\sigma_{f}^{2}{\left(1+\frac{1}{2\alpha }\sum_{m=1}^{B}\frac{{\left(x_{im}-x_{jm}\right)}^{2}}{\sigma_{m}^{2}}\right)}^{-\alpha }$$

This kernel can be interpreted as a combination of exponential quadratic kernels with the mixture parameter *α* > 0 determining the weighting between them. $\sigma_{f}^{2}$ is the scaling factor derived from the total variance. These two are the habitual parameters of the Rational Quadratic Kernel, but in our case, we also allowed feature-dependent lengthscales, i.e., $\sigma_{m}^{2}$.

For training purposes, we assume that the observed variable is formed by noisy observations of the true underlying function *y* = *f*(*x*) + *ϵ.* Moreover, we assume the noise to be additive independently identically Gaussian distributed with zero mean and variance *σ_n_*. Let us define the stacked output values **y** = (*y*_1_,…, *y_n_*)^⊺^, the covariance terms of the test point k* = [*k*(x*, *x*_1_),…, *k*(x*, **x**_*n*_)]^⊺^, and k** = *k*(x*, x*) represents the self-similarity of x*. From the previous model assumption, the output values are distributed according to Equation (6):

(6) $$\left(\begin{array}{c} y\\ f\left(x_{*}\right) \end{array}\right)\sim 𝒩\left(0,\left(\begin{array}{cc} K+\sigma_{n}^{2}I & k_{*}\\ k_{*}^{⊺} & k_{**} \end{array}\right)\right).$$

For prediction purposes, the GPR is obtained by computing the posterior distribution over the unknown output $y_{*},p\left(y_{*}|x_{*},\;𝒟\right)$, where $𝒟\equiv \left\{x_{n},y_{n}|n=1,\ldots ,N\right\}$ is the training dataset. Interestingly, this posterior can be shown to be a Gaussian distribution, $p\left(y_{*}|x_{*},𝒟\right)=𝒩\left(y_{*}|\mu_{{\text{GP}}_{*}},\sigma_{{\text{GP}}_{*}}^{2}\right)$, for which one can estimate the *predictive mean* (point-wise predictions), see Equation (7):

(7) $$\mu_{{\text{GP}}_{*}}=k_{*}^{⊺}{\left(K+\sigma_{n}^{2}I\right)}^{-1}y,$$

and the *predictive variance* (confidence intervals) as in Equation (8):

(8) $$\sigma_{{\text{GP}}_{*}}^{2}=k_{**}-k_{*}^{⊺}{\left(K+\sigma_{n}^{2}I\right)}^{-1}k_{*}.$$

The corresponding hyperparameters ***θ*** are typically selected by Type-II Maximum Likelihood, using the marginal likelihood (also called *evidence*) of the observations, which is also analytical. When the derivatives of the log evidence are also analytical, which is often the case, conjugated gradient ascent is typically used for optimization (see [36] for further details).

In summary, despite being trained with often rather small data sets, GPR models proved to perform well in EO data analysis. GPR even outperformed other non-parametric regression methods, such as random forests (RF) or artificial neural networks (ANN), in remote sensing applications, which may be among others due to the ARD kernel function rendering the model quite flexible. Besides the information about uncertainty, GPR models deliver information about the relevance of bands, which can be used for identifying the sensitive spectral regions [31,37,68].

Note that in our study, we implemented the MATLAB version of GPR models according to Verrelst et al. [12]. In contrast to other programming versions, the MATLAB GPR provides a higher efficiency in the training phase, which leads to lower processing times. A small gain in runtime is essential when using AL methods or processing large scenes within operational setups.

### 2.5 Retrieval with Dimensionality Reduction Strategies

In this section, the two proposed dimensionality reduction approaches are detailed. Specifically, we compared a PCA retrieval strategy (i) against a band ranking procedure (ii). When using PCA (i), spectral data is mapped into a lower-dimensional feature space, which captures most of the variance of the original data. In this way, PCA identifies dominant spectral features but also detects signals in some other bands, depending on the number of considered principal components [21,69]. To obtain the dominant spectral features, PCA solves an optimization problem that seeks to maximize the variance in the transformed space, this is posed under the Rayleigh quotient as:

(9) $$\underset{w}{\arg\;\max}\frac{w^{⊺}\Sigma w}{w^{⊺}w},$$

where Σ is the covariance matrix. The above unconstrained optimization problem (Equation (9)) is equivalent to the following constrained optimization problem:

(10) $$\begin{array}{c} \underset{w}{\arg\;\max}w^{⊺}\Sigma w\\ \text{subject}\mathrm{to}w^{⊺}w=1. \end{array}$$

The solution of the above optimization problem (Equation (10)) can be achieved through the Lagrange multipliers methods, in particular the derived cost function is $ℒ\left(w,\lambda \right)=w^{⊺}\Sigma w-\lambda \left(w^{⊺}w-1\right)$. By computing the partial derivatives, we end up with the equation Σ*w* = *λw*, which requires the computation of the eigenvalues and eigenvectors of the covariance matrix Σ. Σ is a Positive Semi Definite matrix formed by non-negative eigenvalues; these eigenvalues summarize the contribution to the total amount of retained variance by each corresponding eigenvector which are the called principal components of the PCA method. In particular, we follow the criterion based on normalizing the eigenvalues by their total sum. Then, each normalized eigenvalue represents a fraction of the total variance (by summing to one). Our selection rule for the number of principal components is to ensure more than 99.95% of the original variance. To optimally explore the spectral information, at first, we tested the variable estimation accuracy as a function of the total number of PCs. For this purpose, 1 to 25 components were applied to the spectral training data set, GPR algorithms trained, and models run against the in situ data set.

Second (ii), we explored the band ranking procedure. To create the models, we also selected the optimized variable-specific training data sets provided by the AL methodology with the complete CHIME-like spectral setting. We explored a wrapper technique, i.e., feature selection using GPR for automatic band selection, embedded in ARTMO’s GPR-BAT tool. It explores the capability of GPR algorithms to evaluate the predictive power of each available spectral band during the development of a retrieval model. A sequential backward band removal (SBBR) algorithm reveals the bands that contribute most to the development of the model by exploring the automatic relevance determination (ARD) covariance. By eliminating the least contributing band (highest *σ_m_*) and then retraining and validating a new GPR model, the procedure is repeated until, finally only one band remains, indicated by the overall lowest *σ_m_.* Consequently, this routine eventually leads to the identification of the optimal band setting for the variable under consideration.

Therefore, information about the spectral relevance of each band was obtained through the parameter *σ_m_* of the ARD kernel (see Equation (5)), which is the kernel width assigned to the *m*-th band. The *σ_m_* parameter is inversely proportional to the relevance of the band, as it measures the uncertainty of the model with that particular band (highest value means higher uncertainty). To provide a direct relation between *σ_m_* and its relevance, we converted as proposed by [70], and we refer to the value of relevance for each band as *r_m_,* as follows:

(11) $$r_{m}=100\left(1-\frac{\sigma_{m}^{2}}{\max_{\left\{1\leq m\leq B\right\}}\;\sigma_{m}^{2}\Sigma_{m=1}^{B}\;\sigma_{m}^{2}}\right).$$

In addition, to ensure a robust identification of the most sensitive bands and to ensure the inclusion of all simulated samples for validation, the method was combined with k-fold cross-validation (CV) sub-sampling scheme. Specifically, a 3-k sub-group sampling strategy was pursued. Goodness-of-fit validation statistics were averaged for the k validation subsets, i.e., $R_{CV}^{2}$, *RMSE_CV_*, *NRMSE_CV_*, as well as associated SD and min−max rankings. Based on k repetitions, the generated *σ_b_* were k times ranked. A detailed description of the GPR-BAT procedure can be found in Verrelst et al. [31].

### 2.6 Experimental Sites

**Figure 2:**
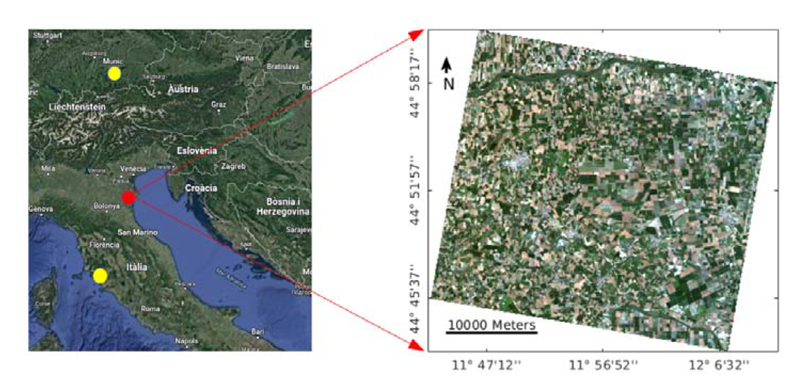
Zoom-in with PRISMA scene at the test site Jolanda di Savoia, Italy. The Grosseto and MNI test sites are also indicated as yellow dots.

The dataset explored in our study was collected during two different campaigns (see Figure 2). The first campaign took place in an agricultural site in the North of Grosseto, located in central Italy (N 42°49.78′, E 11°4.21′) during the summer season of 2018. Sampling was performed within two corn (*Zea mays* L.) fields of varying phenological cycles due to different sowing dates (i.e., early May and mid of June, respectively). The data were collected from 2−7 July and 31 July−1 August 2018 at homogeneous elementary sampling units (ESUs) of 10 × 10 m^2^. LAI was measured at 87 ESUs using either an LAI-2200 plant analyser (LI-COR Biosciences, Lincoln, NE, USA) or a digital hemispherical camera (Nikon CoolPix 990, Tokyo, Japan) equipped with a fish-eye lens (Nikon FC-E8 8 mm, Tokyo, Japan). The LAI-2200 measurements were carried out at the ESUs, repeating one above and four below canopy readings. The hemispherical photographs were processed using the CAN-EYE software (https://www.paca.inrae.fr/can-eye/, accessed on 24 September 2021), providing an average estimate of LAI for each ESU. To obtain CCC, measurements of *C_ab_* were performed within 87 ESUs using a SPAD device (Konica Minolta, Tokyo, Japan), taking the last fully expanded leaf (with five readings at each sampled leaf). In addition, we sampled the last fully developed leaf from three plants within 31 of the 87 ESUs. A few samples under chlorosis conditions, not corresponding to any ESU, were as well collected to enlarge variability. *C_ab_* laboratory extractions were performed on a set of three disks with a 2.2 cm diameter sampled at each leaf. The laboratory analysis included homogenization with methanol (Ultra-Turrax, IKA-Werk, Staufen, Germany), followed by repeated centrifugation and cooling at − 20°. After merging of supernates and filtering (0.45 μm PTFE syringe filter) *C_ab_* could be measured. Lab-extracted *C_ab_* values and corresponding SPAD measurements were used to build the SPAD-*C_ab_* relationship obtaining *R*^2^ = 0.93. The study of Candiani et al. [52] provides in detail the entire laboratory procedure, including resulting equations. This high agreement between SPAD and destructive measurements confirms our choice of the measurement device. However, it must be remarked that SPAD shows some sensitivity towards leaf thickness, which differs between cultivars, developmental stages, and environmental conditions. Nonetheless, several comparative studies found similarly high correlations encouraging the usage of SPAD for in-field sampling [71,72]. Calculation of final *C_ab_* measurements was based on the empirical relationship between the destructive *C_ab_* measurements and the SPAD readings (see also [52]). Finally, LAI was used to upscale the leaf trait to the canopy-level (i.e., CCC in [g/m^2^] = LAI × *C_ab_* × 10^−2^). Measured canopy water content was calculated using LAI and *C_w_* (i.e., CWC in [g/m^2^] = LAI × *C_w_* × 10^4^), which was destructively measured along with *C_m_* within 31 ESUs. Hereby, leaf disks with a 2.2 cm diameter were collected from three corn plants at each ESU and weighted before and after oven-drying (80 ° C for 48 h) using an analytical balance (0.0001 g sensitivity). The two leaf traits were then calculated according to: *C_w_* = (*W_f_* − *W_d_*)/Area; *C_m_* = *W_d_*/Area, where *W_f_* and *W_d_* are fresh and dry weights, respectively.

Simultaneously to the variable sampling, two airborne hyperspectral acquisitions were performed on 7 July and 30 July 2018 in clear sky conditions using the HyPlant DUAL sensor. The sensor covers a spectral range from 380 to 2530 nm (629 bands) with FWHM of 3−10 nm; and provides a ground sampling distance (GSD) from 1 m (7 July 2018) to 4.5 m (30 July 2018). HyPlant raw images were geometrically and atmospherically corrected to top-of-canopy reflectance through a dedicated processing chain described in Siegmann et al. [73].

Data from a second campaign were explored, where measurements were performed at an agricultural test site located in the North of Munich, Southern Germany (N 48°16′, E 11°42′). The long-term consolidated Munich-North-Isar (MNI) site is surrounded by communal farmlands owned by the city of Munich. In the last years, the agricultural test site has been established as a validation site for preparing agricultural algorithms in the context of the German hyperspectral EnMAP mission. The dataset was collected in the growing seasons of 2017 and 2018 of winter wheat (*Triticum aestivum* L.) and corn (*Zea mays* L.). Biophysical and biochemical crop variables were sampled simultaneously with field spectroscopic measurements. Detailed descriptions of the MNI site along with visual documentation can be found in the studies by Berger et al. [20], Danner et al. [74], Wocher et al. [75].

At two fields, a 30 × 30 m^2^ area (according to EnMAP GSD) was defined containing nine ESUs of 10 × 10 m^2^. LAI measurements, in [m^2^/m^2^], were performed with the LI-COR Biosciences LAI-2200 device. Hereby we collected seven below and one above canopy readings and then repeated them twice at each ESU. Finally, the average of all measurements over the nine ESUs was calculated. Measurements of *C_ab_*, in [μg/cm^2^], were collected with a Konica-Minolta SPAD-502 handheld instrument (5 leaves per ESU) at different heights of the crops. To obtain *C_ab_* from SPAD values, a calibration formula was applied obtained from destructive measurements performed at prior campaigns at the MNI site. To achieve this, coefficients of Lichtenthaler [76] were used to estimate *C_ab_* from the SPAD samples [77].

In addition, destructive sampling was performed at each date to determine *C_w_* and *C_m_.* For this, several leaves were cut at each ESU, then weighed, closed in bags, and transported to the laboratory. An LI-COR Biosciences LI-3000C scanner attached to the LI-3050C conveyor belt accessory was employed to measure the leaf area of all samples. *C_w_,* in [cm] equivalent water thickness, and *C_m_,* in [g/cm^2^], were calculated from the mass difference (per unit leaf size) of sample leaves before and after oven-drying at 105 °C (minimum of 24 h) to constant weight.

As for the Grosseto measurements, leaf traits were upscaled to the canopy level by multiplication with LAI. SLA in cm^2^/g was finally obtained by calculating 1/*C_m_* for both campaigns. Table 2 provides an overview of the measured (and calculated) variables from Grosseto and MNI site, with mean values, standard deviations, range, and number of samples. From Grosseto, we have a total of 31 measurements from SLA and CWC and 87 from LAI and CCC. From the MNI site, 28 samples were available for all four variables.

Note that in both campaigns, the optical LAI-2200 instrument was used, which provides an indirect estimate of LAI based on canopy gap fraction following the Beer-Lambert law [78]. Hence, the resulting measurements rather refer to the effective LAI [79,80]. Moreover, the contribution of stalks and fruits or non-photosynthetic biomass may be seen by the instrument. Thus, the obtained values correspond to the effective plant area index [81]. To keep consistency with other studies, we will use the term “LAI” throughout the manuscript.

### 2.7 PRISMA Imagery Acquisition and Pre-Processing

In this study, we explored the data provided by scientific precursor PRISMA of the Italian Space Agency (ASI). PRISMA is a push-broom imaging spectrometer with 240 wavebands providing contiguous spectral information from 400 to 2500 nm, with a nominal spectral sampling interval < 11 nm and an FWHM < 15 nm. The 240 bands are resolved on 1000 across-track pixels with a 12-bit radiometric resolution. PRISMA has a ground spatial resolution of 30 m and a swath width of 30 km. The spacecraft has a body pointing capability, which allows off-nadir observations up to ±14.7°.

For the current study, one PRISMA image was selected, acquired on 26 June 2020 over the agricultural area of Jolanda di Savoia, Italy. The L2D PRISMA reflectance cube was downloaded from the ASI PRISMA mission portal in HDF5 format and read using the prismaread tool [82]. The at-surface reflectance cube was pre-processed to remove artifacts and obtain smooth reflectance spectra. Pre-processing was performed pixel-wise with the R software [83]. In a first step, spikes occurring along track were filtered using the findpeaks function of the pracma package using a threshold of 0.018. In a second step, the spectral regions located within atmospheric gaseous absorption were excluded, as anomalous spikes and dips occurred. These corrections were performed comparing to in situ canopy reflectance spectra collected simultaneously to the PRISMA acquisitions, with wavelengths located at 535−550 nm, 755−780 nm, 755−775 nm, 810−855 nm, 885−970 nm, 1015−1050 nm, 1080−1165 nm, 1225−1285 nm, 1330−1490 nm, 1685−1700 nm, 1725−1750 nm, 1780−1960 nm, and 1990−2030 nm. In a third step, all remaining spectral bands were interpolated using the SplineSmoothGapfilling function included in the FieldSpectroscopyCC package [84]. Finally, we removed atmospheric water absorption domains, i.e., around 1350−1510 nm and 1795−2000 nm, and also the rather noisy bands from 2320 nm onwards. The final PRISMA cube contained 170 spectral bands ranging from 400 to 2320 nm. Correction of PRISMA spectra was also illustrated by Verrelst et al. [12] (see Figure 2), along with the corresponding in situ spectral measurements. For details of the spectral corrections, please refer to Tagliabue et al. [51].

Both the simulated (training) and measured (validation) data sets as well the PRISMA image were spectrally resampled to CHIME-like bands, according to theoretical Gaussian spectral response functions with 10 nm bandwidth. Depending on the quality of the spectral ground measurements and the PRISMA scene, several bands were removed due to noise, as described above. Finally, the spectral datasets contained 198 (for SLA and CWC) or 235 (LAI, CCC, FVC, FAPAR) spectral bands, respectively.

## 3 Results

### 3.1 Active Learning Performance

**Figure 3:**
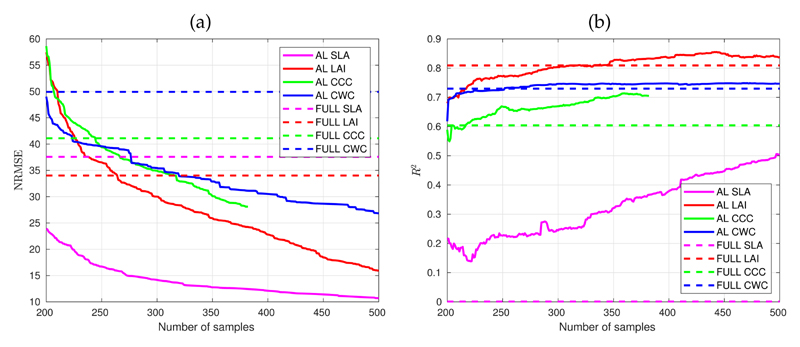
(**a**) NRMSE obtained when applying the EBD procedure to optimize sampling data for estimation of all variables and (**b**) resulting R^2^ of the EBD procedure (AL: optimization with AL, FULL: all samples).

An essential step in developing hybrid models is optimizing the training database, which can be efficiently automated through AL. Figure 3 illustrates the behavior of retrieval performances for all six traits applying the EBD AL procedure run against the merged Grosseto and MNI in situ data set. In Figure 3a, the NRMSE reveals a gradually decreasing trend with an increasing number of samples. This was to be expected, given that using AL, samples are only added if prediction accuracy increased, as evaluated against in situ data. Remarkably, the AL strategy achieved superior accuracy for all the examined variables instead of the models trained with the full data pool. For instance, the EBD reduced data set produced already with 250 samples with the same performance as the full version (with 2000 samples) for LAI and CCC. For CWC, and especially SLA, superior performances were achieved even from the initial 200 samples. NRMSE continued to decline for all variables when adding successful samples. All variables show a gradual decline, although, after about 300 samples, the shape of the SLA curve slowly starts saturation showing a lower benefit in error terms when increasing the number of samples. Overall, the error reduction for SLA is about 15%, while it is about 45A similar pattern of AL effects can be seen in Figure 3b using *R*^2^. Although following the same trend as NRMSE, the *R*^2^ sequence is less smooth than the NRMSE profiles because RMSE was chosen as the internal AL selection criterion. The *R*^2^ is not necessarily behaving the same as RMSE since it rather describes how well the predictor variables (i.e., reflectance) can explain the variation in the response variable (i.e., trait), whereas the RMSE informs how well a model predicts the value of the response variable in absolute terms.

For all variables, the AL procedure led to superior results compared to using the full data sets for model training. We decided on a stopping criterion at 500 samples, providing moderate (CWC, CCC) to high (SLA, LAI) accuracy for the four variables. In the particular case of CCC, the AL procedure already converged with 383 samples, as including any other sample in the model failed to improve the retrieval accuracy. Therefore, our AL optimized dataset was reduced to 500 samples for the variables SLA, LAI, and CWC and 383 samples for CCC. For both FAPAR and FVC variables, in situ data were not available. Thus, a conservative strategy was pursued to build the models by randomly selecting 1000 samples from the SCOPE simulated data sets. This strategy considerably reduced the computational cost and allowed one to maintain the accuracy of the models, guaranteeing robust and optimal performances. Altogether, thanks to AL, the training databases were reduced to more representative datasets leading to winning in both computational execution time and superior accuracy of the trained models. The following step was to add the 26 non-vegetated spectra to the reduced training datasets to ensure that the models are generally applicable to full heterogeneous images.

### 3.2 Optimizing GPR-20PCA and GPR-20BR Retrieval Models

**Figure 4:**
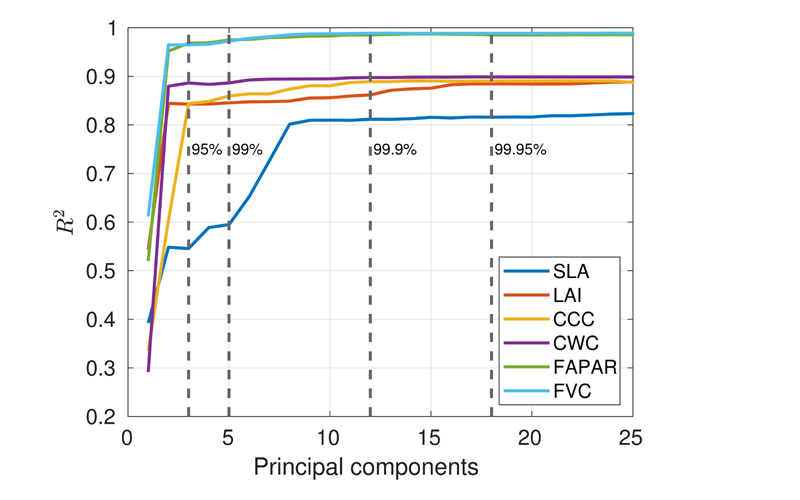
Theoretical retrieval accuracy (*R*^2^) for all six variables achieved by GPR-20PCA models as a function of the number of components, shown from one to 25 (afterward, no more change is visible). A random training-testing data split of 70−30% was applied. Vertical lines represent the traits-averaged cumulative variance covered by the principal components at 95%, 99%, 99.9%, and 99.95%.

Given the traits-specific reduced training datasets complemented by non-vegetated spectra, we subsequently evaluated two spectral dimensionality reduction strategies. Figure 4 provides the theoretical estimation results both in terms of accuracy (*R*^2^) and originally retained variance (vertical dashed lines) as a function of the number of components. Accuracy curves suggest that most variables would sufficiently be estimated by about 16 PCs. Also, the cumulative variance of the principal components, given as vertical lines, reaches 99.95% of the original variance with 18 principal components. To keep the most relevant spectral information, we decided on a final number of 20 PCs assuring optimal results over all variables. Therefore, a PCA with 20 components was applied to the AL-reduced spectral training database for each targeted variable and the final models were named “GPR-20PCA”.

With respect to the BR strategy, the SBBR procedure was applied with 3-fold cross-validation, obtaining a final number of 20 optimal bands to provide a fair comparison with the PCA strategy results. The models were then named “GPR-20BR”. Table 3 illustrates the results for CCC. Goodness-of-fit statistics, i.e., *R*^2^, standard deviation (SD), minimum (min), and maximum (max) are demonstrated for using all 235 bands, 20 and from 15 onwards until eventually only one band is left. The SBBR procedure was applied to all traits and results of optimal band settings were stored.

A summary of the 20-band setting for each trait is given in Table 4. Inspecting the selected wavelengths, they cover the entire spectral-domain provided by CHIME, ranging from 498 nm (for CWC and FAPAR), or at least 813 nm (FVC), until 2136 nm (SLA, CWC, FVC) or 2346 nm (LAI, FAPAR). Hence, essential information is to be found in the visible, near-infrared but also shortwave infrared for retrieval of the targeted variables. The 20 optimal bands were used to compose the training data sets for building trait-specific GPR-20BR models.

### 3.3 Validation of Crop Traits Models

Next, the GPR-20PCA and GPR-20BR models’ performance was validated against the in situ data coming from the MNI and Grosseto campaigns. Table 5 summarizes the goodness-of-fit statistics. To evaluate the added value of these spectral optimization strategies, also results are added when directly entering all bands into the GPR algorithm. Overall, results of both approaches are alike, yet the GPR-20PCA models provided higher accuracy for all six variables. In respect to training times, both models were trained fast, in the order of seconds. Regarding testing time, the GPR-20BR approaches run about two times faster, to be explained by the additional PCA transformation prior to the model training in the case of GPR-20PCA models. Further, for the majority of variables both strategies yielded superior accuracies as opposed when directly using all bands. This underlines the importance of combining hyperspectral data with dimensionality reduction when training MLRAs, such as GPR. Only for CCC superior accuracies are obtained when directly using all bands.

**Figure 5:**
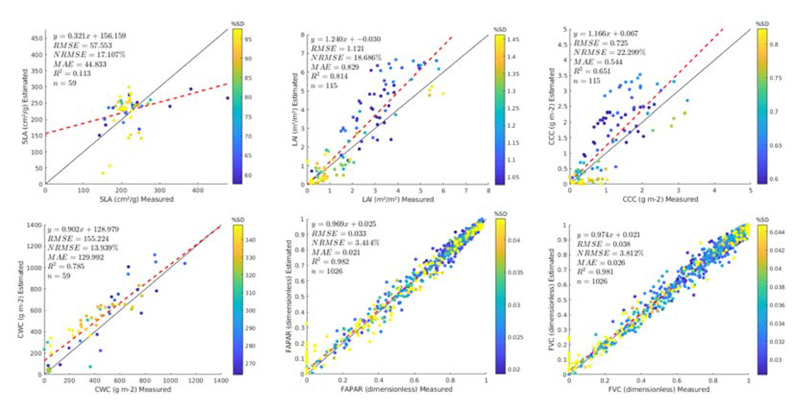
Scatter plots displaying the GPR-20PCA model results against the Grosseto and MNI in situ measurements, with goodness-of-fit statistics. In the case of FAPAR and FVC, theoretical results are provided. The colors of points represent the standard deviation (SD) obtained by the GPR models.

**Figure 6:**
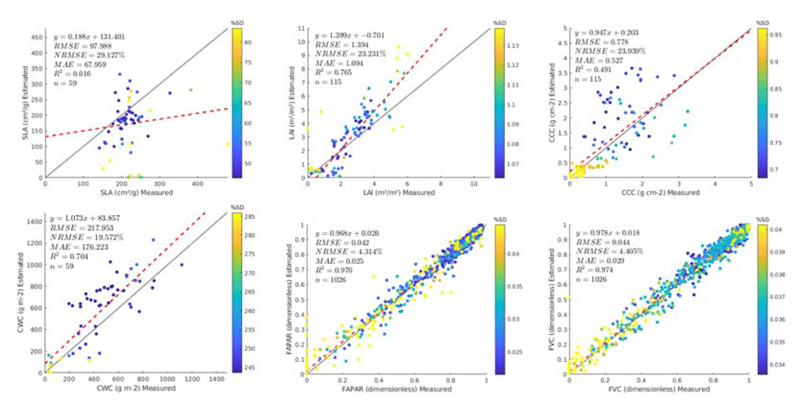
Scatter plots displaying the GPR-20BR model results against the Grosseto and MNI in situ measurements, with goodness-of-fit statistics. In the case of FAPAR and FVC, theoretical results are provided. The colors of points represent the standard deviation (SD) obtained by the GPR models.

Results of the GPR-20PCA and GPR-20BR strategies are also shown as scatter plots in Figures 5 and 6, respectively. The scatter plots provide some additional information, such as the relative uncertainty, expressed as percentage of coefficient of variation (CV: SD/mean estimate) and the linear regression function. The following main trends must be remarked. The SLA models led to poorest validation results (17.11% for GPR-20PCA, and 29.1% for GPR-20BR).

It must be remarked that adding non-vegetated spectra to the AL-optimized dataset and re-training the models degraded the results (from NRMSE = 11%, see also Figure 3). Degradation of validation results after adding bare soil or other non-green spectra has been observed before [12,21], yet it is an essential step to render models generally applicable, i.e., able to interpret non-vegetated surfaces correctly. The canopy variables LAI, CCC, and CWC yielded more consistent results and aligned with the AL optimization. Close-to-zero estimates typically go along with higher relative uncertainties (in part due to the near-zero estimate with some SD around it). However, LAI and CCC estimates provide, in general, low uncertainties. CWC led to higher uncertainties with the PCA strategy but not with the BR strategy, suggesting that the latter showed more confidence in the estimates despite its poorer validation result (GPR-20BR, NRMSE = 19.6% vs. GPR-20PCA, NRMSE = 13.9%). Finally, FVC and FAPAR yielded the best results, although no validation data was available for these variables. Hence, only theoretical validation can be presented.

In Appendix A Table A1 we further provide the results of retrieval models built with the variable-specific optimized band combination and validated against the same in situ data sets as presented in Table 5. The optimized number of bands ranged from two (for CWC) to 227 (for CCC) and results slightly improved compared to models based on 20 optimal bands. However, for most variables, the GPR-20PCA models outperformed all band ranking strategies. Hence, in summary, these statistics suggest that a slight preference goes towards the PCA strategy; yet both models produced estimates with low-to-high uncertainties for all variables.

### 3.4 PCA vs. BR Analysis: Polar Plots

**Figure 7:**
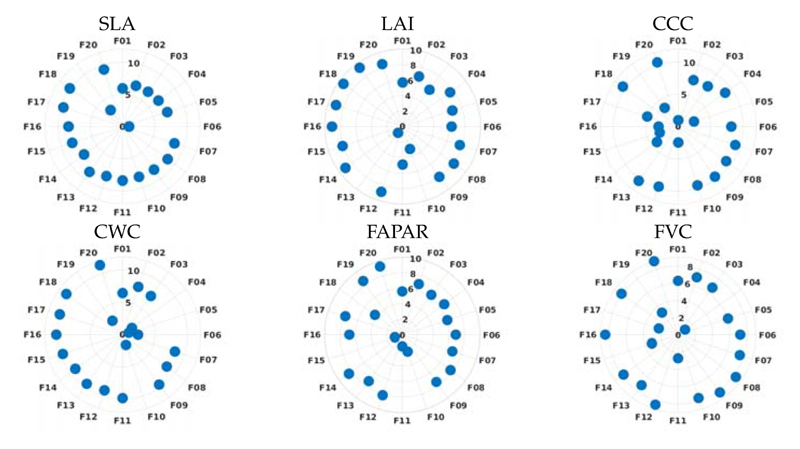
Polar plots for each variable using the GPR-20PCA models. All 20 components are displayed around the circumference. Distance to origin represents the importance of each component: the more outside, the more important.

Following the development of the two types of hybrid models for the targeted crop traits, i.e., based on 20 PCA components (GPR-20PCA) and based on 20 best-selected bands (GPR-20BR), we inspected the contribution of the 20 features for building the final GPR models. The feature relevance can be demonstrated in a polar plot according to Equation (11), i.e., the more positioned to the outside, the more relevant. Figure 7 visualizes the relevance of 20 PCAs for the six hybrid models. Notably, the first component provides significant relevance, but the most important features are located in higher components. Moreover, the following components show less impact in building up a prediction model towards the targeted variable. Overall, relevant components are to be found from the 7th (e.g., SLA) onwards. For LAI, we found most information in 8th, 9th, and higher components (i.e., 14th−20th, whereas the most relevant components for CWC are located from the 11th onwards. Moreover, in the case of CCC, FAPAR and FVC, rather higher components provide the most weight in building the regression model. Hence, we conclude that higher components tend to provide the required subtle information necessary for constructing trait-specific retrieval models.

**Figure 8:**
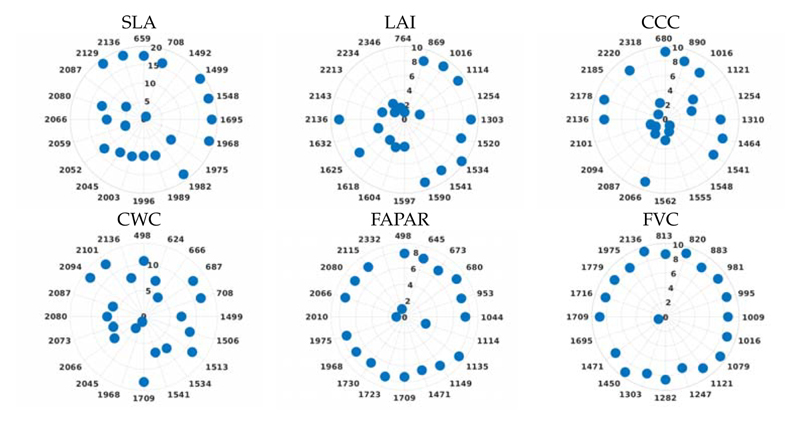
Polar Plots for each variable using the GPR-20BR models. All 20 best-selected bands (in nm) are displayed around the circumference. Distance to origin represents the importance of each band: the more outside, the more important.

Likewise, Figure 8 visualizes the relevance of the 20 most sensitive bands extracted according to GPR-BAT for the six hybrid models. Thus, each polar plot represents the importance of 20 selected bands for a specific variable. Hereby, it is of interest to inspect the relevance of each band according to its sensitivity toward specific variables. For instance, LAI and FVC are structural variables, thus driven by optical properties, position, and density of the leaf elements, as well as the soil background. CCC and CWC are LAI-combined canopy variables with leaf variables (*C_ab_* and *C_w_*); thus, here, both the role of LAI and the leaf variables drive the band sensitivity. Finally, FAPAR and FVC are also closely related to LAI as they are driven by the amount and position of the green leaves. The leaf variable SLA extracted the majority of important bands in the visible (526−715 nm) and then added one band in the near-infrared (NIR) (1072 nm) and two bands in the shortwave infrared (SWIR) (1709, 1968 nm). In particular, the sensitivity towards the SWIR can be explained by pronounced absorption features of cellulose and lignin in this domain, being constituents of SLA (or *C_m_*). When inspecting the 20 selected bands for LAI, they fell in the 638−1303 nm range only. Analysis for CCC identified the same or neighboring bands with the difference of a dominant band in the blue visible (498 nm). Regarding CWC, the 20 best bands are spread all along with the visible to NIR (VNIR) domain, including the water absorption regions. FAPAR follows a strategy of bands throughout the entire VNIR range, starting from a band in the blue, a few in the red, and then most bands in the NIR and SWIR. The FVC analysis selected the first band at 813 nm, followed by sampling throughout the NIR and SWIR. As FVC is driven by the relationship between vegetation cover and soil underneath, typically, the spectral profile of vegetation and soil contrasts the most in the SWIR.

### 3.5 Mapping Crop Traits Using CHIME-like Imagery and Comparison

**Figure 9:**
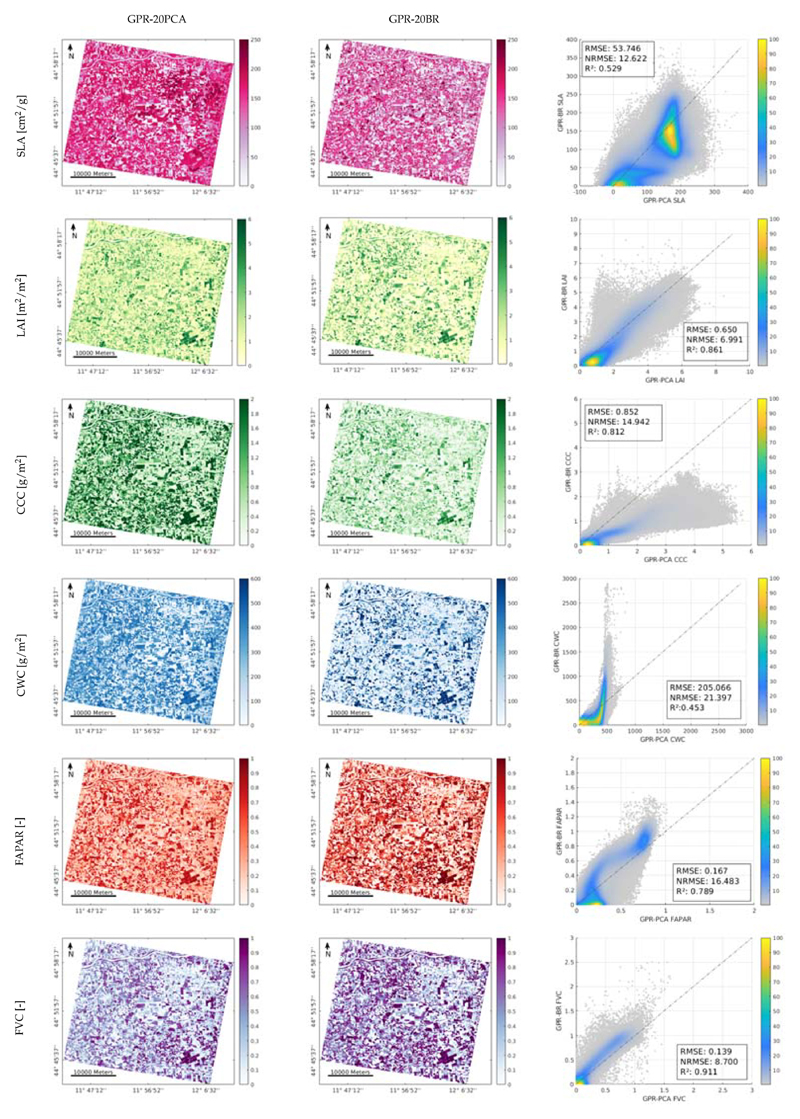
Mapping results of estimated variables SLA, LAI, CCC, CWC, FAPAR and FVC over Jolanda di Savoia site on 26 June 2020. The PRISMA scene was spectrally resampled to future CHIME configuration. Maps of the GPR-20PCA (**left**) GPR-20BR (**right**) generated models, and comparison of the two methodologies as scatter plots (**right**).

As a final step, we applied the GPR-20PCA and GPR-20BR models to a PRISMA image over the Jolanda di Savoia site that was resampled to CHIME band settings. The full image was processed by the two models as demonstrated in Figure 9, allowing us to evaluate whether vegetated land, as well as non-vegetated surfaces, were correctly processed. Maps for the two approaches were generated and compared using a scatter plot (see Figure 9, right), revealing some trends and differences. For instance, the cropland trait maps show pronounced values over vegetated areas. At the same time, zero or close-to-zero values were obtained over non-vegetated surfaces, such as the river or over bare soils, man-made surfaces, or senescent fields. However, when interpreting the mapping over vegetated surfaces combined with the validation results, the SLA maps provided the lowest accuracy, as both GPR-20PCA and GPR-20BR models led to low validation statistics (see Table 5). The SLA GPR-20PCA map also shows pronounced higher values, as confirmed by the scatter plot. The LAI maps emerged among the most consistent maps, with similar mapping results for both GPR-20PCA and GPR-20BR approaches, and confirmed by the scatter plot. Larger differences between both model approaches were generated for the variables CCC and CWC. In the case of CCC, the GPR-20PCA model shows systematic overestimation as opposed to GPR-20BR. Yet, as the GPR-20PCA model was validated as more accurate, it suggests that rather the GPR-20BR approach led to underestimation. Most pronounced differences can be observed for CWC, with the production of out-of-range values for the GPR-20BR model, as also visible in the scatter plot. Regarding FAPAR and FVC, both models retrieved estimates within the expected 0−1 range, although in the case of the FAPAR systematic differences emerged with GPR-20PCA giving more emphasis to lower values than GPR-20BR. From all variables, the most consistent maps were achieved with FVC, whereby the two maps closely matched with *R*^2^ of 0.93.

The mapping runtime was recorded as processed on a personal computer (Ubuntu 20.04 LTS 64-bits OS, Intel i7-9700K CPI 3.60 GHz, 32 GB RAM). Runtime can become an important bottleneck when it comes to operational processing. Optimization in both sampling and spectral domains allows fast processing and ensures lightweight models. While both models rely on 20 features, in the case of GPR-20PCA, an additional step of PCA conversion is introduced. This leads, on average, to 10% slower processing with the GPR-20PCA models of the CHIME-like image, with an overall runtime of 45 s versus 40 s in the case of GPR-20BR. If all available CHIME bands were used, it would not only lead to poorer results but also to substantially longer runtime: a model built with all bands needs on average 418 s to process the full scene, which is 10.4 and 9.3 times slower than GPR-20PCA and GPR-20BR models, respectively.

## 4 Discussion

We analyzed the role of dimensionality reduction methods within hybrid retrieval models applicable to hyperspectral data. In the following, we discuss the key aspects of the pursued strategies, being: (1) the role of active learning in optimizing training samples, (2) the role of dimensionality reduction strategies in spectral domain, (3) implications in preparation for CHIME, and finally (4) challenges and opportunities.

### 4.1 Role of Active Learning in Optimizing Training Samples

A first key result is the substantially improved accuracy achieved thanks to applying the AL strategy as opposed to using full non-optimised training datasets. Due to the hybrid nature of the method, AL adapts the RTM simulated training data sets to real world situations by tuning them towards in-field reference data, still keeping independence through randomly selecting initial training data (10% of the 2000 simulations). Here it is assumed that sufficiently generic models are processed since reference data came from two campaigns, covering a variety of crop conditions. By initiating the AL sequencing with a random pool of 200 samples, in total the models were finalized with about 500 samples, since this number was decided here as stopping criterion. As also demonstrated by prior studies, the specific procedure with AL allows to build lightweight yet accurate retrieval models, which still retain independence and generality [21,32,34,85,86]. These studies as well as our results underline that training datasets based on simulations can be automatically optimized making use of AL strategies, thereby suggesting that the quality plays a more important role than the quantity of the training data. In other words, to generalise the models well, it is crucial that the training data are an accurate representation of the full variability found in nature. Even if large training samples are available, they can be non-representative in case the sampling selection method was flawed (sampling bias), which is avoided by using AL heuristics. When mapping full scenes, which are usually characterized by diverse land covers, it must be ensured that the retrieval models are able to recognize multiple spectral surfaces. This adaptation can be obtained as applied here, i.e., by adding diverse non-vegetated spectra to the AL-optimized training samples, e.g., coming from bare soil, water, or man-made surfaces. Providing training datasets with such additional spectra from the hyperspectral satellite scenes is an important step for generating generally applicable hybrid retrieval models and processing different cultivated landscapes into vegetation trait maps (e.g., refs. [12,23]).

### 4.2 Role of Dimensionality Reduction Strategies in Spectral Domain

Seeking for efficient reduction in the spectral domain was the following step in the process optimization. Here we compared the performance of feature transformation (PCA) against a feature extraction (band selection) method. For all six considered variables, evaluation with the in situ data sets achieved superior estimation accuracy for GPR-20PCA models than for GPR-20BR models. The reason for the superior results of the feature transformation approach can be found in the inherent nature of PCA, where the complete spectral information is converted into a defined number of unique components. In this way, a richer dataset is available for GPR algorithm training than when selecting a few bands only. In our analysis we standardized the number of components and bands to 20, allowing for a fair comparison between both approaches. Nonetheless, model performances may still be improved when optimizing the number of components for each variable individually. Although Figure 4 suggests that including more than 20 components within the training phase will hardly alter the GPR models’ performance, adding higher components (i.e., >20) may provide some extra relevant subtle information [21,87], yet it also comes with the risk to include rather noise [88].

Instead, selecting the optimal number of bands according to the SBBR strategy would allow a distinct variable-specific optimization. While the 20 best selected bands provided a good overall accuracy, they may not be top-performing. Adding more or less well-chosen bands through the SBBR method may further improve the model performance depending on the variable (see also Appendix A Table A1). Comparison of both strategies revealed that still some improvements can be gained as opposed to using 20 bands, although increase in accuracy was minimal. For instance, the relative errors as expressed by NRMSE are of the same order as for the 20 best bands for SLA, FAPAR and FVC. Some improvements could be achieved for LAI, however, for CWC, the 2-bands model performed poorer. Accordingly, this suggests that the optimal number of bands as evaluated by the SBBR strategy does not necessarily lead to the best models when validated against in situ data. While the runtime is most efficient, models built on a few bands may be unable to keep the same quality when applied to external data in an operational mapping context. Altogether, the selection of a standard variable-specific 20-best band setting can be considered a robust strategy—yet bearing in mind that superior results are achieved by PCA transformation strategies.

Despite the overall superior performances achieved by GPR-20PCA models, a benefit of using individual band optimization strategies is the possibility of interpretation in view of their sensitivity towards the targeted variables. For instance, selected bands can be compared against a global sensitivity analysis (GSA) run over the input-outputs of a leaf-canopy RTM, e.g., PROSAIL [89]. Based on GSA results, the contribution of the different input variables to the overall spectral output (e.g., reflectance) can be quantified and used as a framework to interpret the outputs of the GPR-20BR models. Using a GSA, we can identify the prime driving variables of spectral reflectance. As demonstrated by previous studies, up to 40% LAI explains most of the total variability, especially from the NIR onwards [89,90]. This also led to the selection of bands located in the NIR in the case of upscaled leaf variables, such as CCC (1310,1464, 1541 nm and some bands in the SWIR beyond 2000 nm). Besides identifying the driving variables of the vegetated canopy, we can also see spectral transition zones for specific variables, reflected by the 764 nm band for LAI (see Figure 2 in Berger et al. [57]), or by the 1968 nm band for CWC (see Figure 3 in Verrelst et al. [90]).

Direct band-related interpretation is impossible for feature engineering techniques where the original spectral information is transformed into components. However, using PCA, we preserve the statistical variability of the spectral information providing crucial information for retrieving the multiple vegetation traits [50]. In previous hyperspectral studies [23,27,91], PCA-based methods were also more successful in retrieving different vegetation traits than band-related approaches (e.g., using ratio band vegetation indices). Further improvement of the models’ robustness can be achieved by injecting artificial noise into the spectral training data. The rationale is that simulated data is overly perfect as opposed to image data where noise is always present for multiple reasons, e.g., due to sensor electronics and optics or poor geometric, radiometric, or atmospheric corrections. Adding noise to the synthetic training data may also support accounting for variability present on the surface, e.g., due to sub-pixel heterogeneity [19,26,92]. It must also be remarked, however, that the optimized sampling through AL techniques largely surpasses the need for adding noise, as was observed in recent active learning studies [12,21]. Here, we also found that the role of noise was negligible (results not shown).

### 4.3 Implications for the Preparation of CHIME

This work was carried out within the framework of ESA’s CHIME E2E mission performance simulator that aims to accurately reproduce all required steps of an EO data processing chain. In the E2E framework, we start with data acquisition, followed by several processing steps and finalizing with surface variable maps, including crop traits as presented here [13]. In the ongoing CHIME preparation phase, the E2E simulator will be further adapted and extended until the launch of the satellite into space [13]. One of the main features of the E2E simulator is its capability to evaluate the products with reference input data, allowing tuning and further improvements of the models by exploring actual campaign datasets [13].

So far, hybrid models exploring CHIME’s E2E data were based solely on the PCA strategy [13,52]. The GPR-20PCA models were evaluated as convenient, as all available spectral information was directly converted into 20 components. However, it remained to be investigated whether this approach provided optimal performance. Comparing the accuracy of the GPR-20PCA to GPR-20BR retrieval models and validating against a representative in situ dataset, our study confirmed the validity of these models: overall, GPR-20PCA models outperformed GPR-20BR for all variables, though for some specific variables, differences were small (FAPAR, FVC). It must also be noted that we explored GPR as a core retrieval algorithm to be implemented into CHIME’s L2B Vegetation module, mainly due to its outstanding predictive performances and capability of providing uncertainties associated with the predictions [38]. Yet, likewise, other promising MLRAs deserve to be evaluated on their retrieval performances and portability (e.g., see review provided by Verrelst et al. [17]). Potentially attractive alternatives would be RF regression or powerful designs of ANNs, with RFs more likely preferred given their ability to calculate associated uncertainties in the form of a quantile RF approach [93].

### 4.4 Challenges and Opportunities

This study was built upon earlier efforts in prototyping new-generation vegetation traits retrieval algorithms in preparation for the upcoming CHIME, see also [12,13,21,51,52]. These preceding studies focused on hybrid retrieval algorithms in combination with PCA. This tendency towards hybrid strategies may be explained by the synergistic usage of complementary methods blending their advantages: (1) the processing speed of data-driven machine learning regression, with (2) physical extrapolation capacities of RTM based modeling, often in combination with (3) dimensionality reduction in the sample and spectral domain. It is expected that this research path will continue to develop, eventually leading to robust models that are globally applicable by the time CHIME is launched. Despite their promising prospects, each used method faces limitations, which could be addressed and improved by future studies. For instance, a critical point to be considered in hybrid model development with AL strategies is that it usually involves tuning against available in situ data sampled at selected sites. At the same time, we aimed to provide sufficiently generic retrieval models applicable worldwide for any time in the year. While here we combined in situ sampled data from two different campaigns and initiated the AL sequence with a random training dataset of 200 samples, the training and validation datasets may still be limited in quality and quantity for developing globally-applicable models. This holds, in particular, true for the estimation of leaf-level traits, where additional work is needed to provide optimized retrieval models. Ideally, the in situ data set covers a broad range of vegetation types collected during multiple phenological stages in combination with spectral data and corresponding uncertainty information of the measurements [14,24,26]. A further critical issue when employing AL is the optimal timing at which learning should be stopped, i.e., the stopping criterion [94]. In a future study, this could be investigated along with the size of the original data pools.

As a closing remark, it should be noted that although the GPR-20PCA strategy ensures the capture of all information within the spectral data, it also faces some drawbacks. First, the PCA processing step takes about 10% additional runtime instead of the GPR-20BR models. Second, converting all bands into components goes along with a risk of including information on noisy bands, affecting training and image data. In this respect, models may perform less accurately when passing through the complete E2E and real processing chain due to the existence of unexpected artifacts within the image after passing atmospheric correction. If noisy bands appear in future CHIME L2A data, a solution could be to exclude those bands in the subsequent retrieval module. An alternative option is to move towards the optimized band selection strategy to ensure that noisy bands are excluded, as was successfully evaluated in this work.

## 5 Conclusions

Recent advances in hyperspectral instrument designs potentially allow accurate quantification of the status and dynamics of crucial crop traits, like SLA, LAI, or CWC, over vast agricultural areas. These unprecedented data streams, as delivered by new-generation and upcoming operational spaceborne imaging spectroscopy missions, such as CHIME, can improve our understanding of physiological processes related to photosynthesis, transpiration and respiration, being the main drivers of crop growth and development.

A workflow was developed to optimize hybrid hyperspectral retrieval models where we first applied reduction in the sampling domain through active learning and then compared two spectral dimensionality reduction strategies, i.e., GPR-20PCA and GPR-20BR. We found that retrieval results of the PCA strategy slightly outperformed those of the band ranking procedure for all considered variables, which may indicate a higher fidelity of the GPR-20PCA models. Besides physical validation using in situ data, demonstrating accurate spatial application is crucial for indirectly evaluating the models’ capabilities. In this respect, both modeling approaches achieved meaningful mapping results over a heterogeneous landscape, including multiple cover types.

Overall, based on these findings, we recommend using GPR-20PCA models as the most efficient strategy for estimating multiple traits from hyperspectral data streams. However, if inconsistent retrieval performances occur, GPR-20BR models are recommended as a backup. With the ambition to pave the way for operational usage within CHIME, we suggest further evaluating the generality of the proposed models in their capability of global coverage processing.

## Supplementary Material

Supplementary Material

## Figures and Tables

**Table 1:** Parameterization of SCOPE and BSM soil reflectance models, with notations, units, ranges and distributions of inputs used to simulate the spectral training database. $\bar{x}$: mean, SD: standard deviation.

	Model Variables	Units	Range (Min-Max)	Distribution
*Leaf Variables*				
*N*	Leaf structure parameter	unitless	1.0−2.7	Gaussian ($\bar{x}$: 1.5, SD: 0.5)
*C_ab_*	Leaf chlorophyll content	[μg/cm^2^]	0−80	Gaussian ($\bar{x}$: 45, SD: 35)
*C_m_*	Leaf dry matter content	[g/cm^2^]	0.002−0.02	Gaussian ($\bar{x}$: 0.0075, SD: 0.005)
*C_w_*	Leaf water content	[g/cm^2^]	0.005−0.035	Gaussian ($\bar{x}$: 0.015, SD: 0.0075)
*C_xc_*	Leaf carotenoid content	[μg/cm^2^]	0−20	Uniform
*Canopy Variables*				
LAI	Leaf area index	[m^2^/m^2^]	0.1−8	Uniform
LIDF	Leaf Inclination	rad	−1−1	Uniform
*α_soil_*	Soil scaling factor	unitless	0−1	Uniform
SZA	Sun zenith angle	[°]	0−80	Uniform
OZA	Observer zenith angle	[°]	0−25	Uniform
RAA	Relative azimuth angle	[°]	0−180	Uniform
*Soil variables*				
*SMC*	Soil Moisture Content	[%]	5−55	Gaussian ($\bar{x}$: 25, SD: 12.5)
*BSM*	BSM Brightness	[%]	0−0.9	Gaussian ($\bar{x}$: 0.5, SD: 0.25)
*BSMlat*	BSM latitude	[°]	20−40	Gaussian ($\bar{x}$: 25, SD: 12.5)
*BSMlong*	BSM longitude	[°]	45−65	Gaussian ($\bar{x}$: 50, SD: 10)

**Table 2:** Overview statistics of measured and targeted variables of Grosseto and MNI campaigns.

Variable (Abr)	Unit	Mean (SD)	Range	No. of Samples
Specific Leaf Area (SLA)	cm^2^/g	219 (51.2)	142−478	59
Leaf Area Index (LAI)	m^2^/m^2^	2.1 (1.6)	0−6	115
Canopy Chloropyll Content (CCC)	g/m^2^	0.97 (0.7)	0−3.2	115
Canopy Water Content (CWC)	g/m^2^	417 (271)	0−1113	59

**Table 3:** An SBBR example of a CCC variable with goodness-of-fit statistics based on 3-fold crossvalidation as run by GPR-BAT.

#Bands	*R* ^2^	SD	Min	Max	Wavelengths (nm)
235	0.869	0.062	0.832	0.940	All bands
⋮	⋮	⋮	⋮	⋮	⋮
20	0.879	0.071	0.825	0.960	680 890 1016 1121 1254 1310 1464 1541 1548 1555 1562 2066 2087 2094 2101 2136 2178 2185 2220 2318
⋮	⋮	⋮	⋮	⋮	⋮
15	0.879	0.071	0.825	0.960	680 890 1016 1121 1254 1310 1464 1541 1548 1555 1562 2136 2185 2220 2318
14	0.879	0.071	0.825	0.960	680 890 1016 1121 1254 1310 1464 1541 1548 1555 1562 2185 2220 2318
13	0.879	0.071	0.825	0.960	680 890 1016 1121 1254 1310 1464 1541 1555 1562 2185 2220 2318
12	0.879	0.071	0.825	0.960	680 890 1016 1121 1254 1310 1464 1541 1555 1562 2220 2318
11	0.883	0.069	0.825	0.960	680 890 1016 1121 1254 1310 1464 1541 1562 2220 2318
10	0.872	0.050	0.825	0.925	680 890 1016 1121 1254 1310 1464 1555 2220 2318
9	0.894	0.050	0.825	0.925	680 890 1016 1121 1254 1310 1464 2220 2318
8	0.874	0.050	0.825	0.925	680 890 1016 1121 1254 1310 1464 2318
7	0.873	0.049	0.825	0.924	680 890 1016 1121 1254 1310 1464
6	0.869	0.044	0.824	0.913	680 890 1016 1121 1310 1464
5	0.851	0.076	0.765	0.913	680 890 1016 1310 1464
4	0.850	0.087	0.757	0.913	680 890 1310 1464
3	0.808	0.091	0.747	0.913	680 890 1310
2	0.796	0.099	0.731	0.910	890 1310
1	0.237	0.193	0.069	0.449	1310

**Table 4:** Optimal band settings composed of the 20 best bands for each variable as identified by SBBR. Selected bands were used to build trait-specific GPR-20BR retrieval models.

#Variable	Wavelengths (nm)																			
SLA	659	708	1492	1499	1548	1695	1968	1975	1982	1989	1996	2003	2045	2052	2059	2066	2080	2087	2129	2136
LAI	764	869	1016	1114	1254	1303	1520	1534	1541	1590	1597	1604	1618	1625	1632	2136	2143	2213	2234	2346
CCC	680	890	1016	1121	1254	1310	1464	1541	1548	1555	1562	2066	2087	2094	2101	2136	2178	2185	2220	2318
CWC	498	624	666	687	708	1499	1506	1513	1534	1541	1709	1968	2045	2066	2073	2080	2087	2094	2101	2136
FAPAR	498	645	673	680	953	1044	1114	1135	1149	1471	1709	1723	1730	1968	1975	2010	2066	2080	2115	2332
FVC	813	820	883	981	995	1009	1016	1079	1121	1247	1282	1303	1450	1471	1695	1709	1716	1779	1975	2136

**Table 5:** Goodness-of-fit statistics against the Grosseto and MNI in situ datasets (and theoretical results for FVC and FAPAR) were achieved with both methodologies, GPR-20PCA and GPR-20BR, and also with all available bands: variables, number of samples (N), RMSE, relative RMSE (RRMSE), NRMSE, *R*^2^, as well as computational time (s: seconds) for algorithm training and model testing.

Variable	N Samples	RMSE	RRMSE	NRMSE	*R* ^2^	Train Time (s)	Test Time (s)
**SLA 20PCA**	526	57.553	26.190	17.107	0.113	8.978	0.005
**SLA 20BR**	526	97.988	44.590	29.127	0.016	6.175	0.009
**SLA all bands**	526	120.151	54.676	35.715	0.095	795.557	0.011
**LAI 20PCA**	526	1.121	53.235	18.686	0.814	7.393	0.003
**LAI 20BR**	526	1.394	66.184	23.231	0.765	5.602	0.009
**LAI all bands**	526	1.272	60.391	21.197	0.598	317.261	0.020
**CCC 20PCA**	409	0.725	74.676	22.299	0.651	3.831	0.003
**CCC 20BR**	409	0.778	80.166	23.939	0.491	21.394	0.023
**CCC all bands**	409	0.586	60.414	18.041	0.715	156.698	0.028
**CWC 20PCA**	526	155.224	37.189	13.939	0.785	6.730	0.005
**CWC 20BR**	526	217.953	52.219	19.572	0.704	5.895	0.003
**CWC all bands**	526	381.125	91.313	34.225	0.595	387.714	0.011
**FAPAR 20PCA**	1026	0.033	4.218	3.413	0.982	21.619	0.032
**FAPAR 20BR**	1026	0.042	5.329	4.313	0.970	13.205	0.014
**FAPAR all bands**	1026	0.056	7.168	5.801	0.948	1842	0.053
**FVC 20PCA**	1026	0.038	4.934	3.812	0.981	26.943	0.022
**FVC 20BR**	1026	0.044	5.700	4.404	0.974	12.709	0.010
**FVC all bands**	1026	0.039	5.113	3.951	0.979	1969	0.093

## Data Availability

Not applicable.

## References

[1] Prosekov AY, Ivanova SA (2018). Food security: The challenge of the present. Geoforum.

[2] Atzberger C (2013). Advances in Remote Sensing of Agriculture: Context Description, Existing Operational Monitoring Systems and Major Information Needs. Remote Sens.

[3] Ustin SL, Middleton EM (2021). Current and near-term advances in Earth observation for ecological applications. Ecol Process.

[4] Loizzo R, Daraio M, Guarini R, Longo F, Lorusso R, Dini L, Lopinto E (2019). Prisma Mission Status and Perspective.

[5] Guanter L, Kaufmann H, Segl K, Foerster S, Rogass C, Chabrillat S, Kuester T, Hollstein A, Rossner G, Chlebek C (2015). The EnMAP Spaceborne Imaging Spectroscopy Mission for Earth Observation. Remote Sens.

[6] Drusch M, Moreno J, Del Bello U, Franco R, Goulas Y, Huth A, Kraft S, Middleton EM, Miglietta F, Mohammed G (2016). The FLuorescence EXplorer Mission Concept—ESA’s Earth Explorer 8. IEEE Trans Geosci Remote Sens.

[7] Board SS (2018). Thriving on Our Changing Planet: A Decadal Strategy for Earth Observation from Space.

[8] Nieke J, Rast M (2019). Status: Copernicus Hyperspectral Imaging Mission For The Environment (CHIME).

[9] Rast M, Painter TH (2019). Earth Observation Imaging Spectroscopy for Terrestrial Systems: An Overview of Its History, Techniques, and Applications of Its Missions. Surv Geophys.

[10] Buschkamp P, Sang B, Peacocke P, Pieraccini S, Geiss MJ, Roth C, Moreau V, Borguet B, Maresi L, Rast M (2021). CHIME’s hyperspectral imaging spectrometer design result from phase A/B1.

[11] Rast M, Ananasso C, Bach H, Ben-Dor E, Chabrillat S, Colombo R, Del Bello U, Feret J, Giardino C, Green RO (2019). Copernicus Hyperspectral Imaging Mission for the Environment: Mission Requirements Document.

[12] Verrelst J, Rivera-Caicedo JP, Reyes-Muñoz P, Morata M, Amin E, Tagliabue G, Panigada C, Hank T, Berger K (2021). Mapping landscape canopy nitrogen content from space using PRISMA data. ISPRS J Photogramm Remote Sens.

[13] Verrelst J, De Grave C, Amin E, Reyes P, Morata M, Portales E, Belda S, Tagliabue G, Panigada C, Boschetti M (2021). Prototyping vegetation traits models in the context of the hyperspectral CHIME mission preparation.

[14] Hank TB, Berger K, Bach H, Clevers JG, Gitelson A, Zarco-Tejada P, Mauser W (2019). Spaceborne imaging spectroscopy for sustainable agriculture: Contributions and challenges. Surv Geophys.

[15] Weiss M, Jacob F, Duveiller G (2020). Remote sensing for agricultural applications: A meta-review. Remote Sens Environ.

[16] Verrelst J, Camps-Valls G, Muñoz Marí J, Rivera J, Veroustraete F, Clevers J, Moreno J (2015). Optical remote sensing and the retrieval of terrestrial vegetation bio-geophysical properties—A review. ISPRS J Photogramm Remote Sens.

[17] Verrelst J, Malenovský Z, Van der Tol C, Camps-Valls G, Gastellu-Etchegorry JP, Lewis P, North P, Moreno J (2019). Quantifying vegetation biophysical variables from imaging spectroscopy data: A review on retrieval methods. Surv Geophys.

[18] Verrelst J, Vicent J, Rivera-Caicedo JP, Lumbierres M, Morcillo-Pallarés P, Moreno J (2019). Global Sensitivity Analysis of Leaf-Canopy-Atmosphere RTMs: Implications for Biophysical Variables Retrieval from Top-of-Atmosphere Radiance Data. Remote Sens.

[19] Brede B, Verrelst J, Gastellu-Etchegorry JP, Clevers JG, Goudzwaard L, den Ouden J, Verbesselt J, Herold M (2020). Assessment of workflow feature selection on forest LAI prediction with sentinel-2A MSI, landsat 7 ETM+ and Landsat 8 OLI. Remote Sens.

[20] Berger K, Verrelst J, Féret JB, Hank T, Wocher M, Mauser W, Camps-Valls G (2020). Retrieval of aboveground crop nitrogen content with a hybrid machine learning method. Int J Appl Earth Obs Geoinf.

[21] Berger K, Hank T, Halabuk A, Rivera-Caicedo JP, Wocher M, Mojses M, Gerhátová K, Tagliabue G, Dolz MM, Venteo ABP (2021). Assessing Non-Photosynthetic Cropland Biomass from Spaceborne Hyperspectral Imagery. Remote Sens.

[22] Danner M, Berger K, Wocher M, Mauser W, Hank T (2021). Efficient RTM-based training of machine learning regression algorithms to quantify biophysical & biochemical traits of agricultural crops. ISPRS J Photogramm Remote Sens.

[23] De Grave C, Verrelst J, Morcillo-Pallarés P, Pipia L, Rivera-Caicedo JP, Amin E, Belda S, Moreno J (2020). Quantifying vegetation biophysical variables from the Sentinel-3/FLEX tandem mission: Evaluation of the synergy of OLCI and FLORIS data sources. Remote Sens Environ.

[24] Salinero-Delgado M, Estévez J, Pipia L, Belda S, Berger K, Paredes Gómez V, Verrelst J (2021). Monitoring Cropland Phenology on Google Earth Engine Using Gaussian Process Regression. Remote Sens.

[25] Estévez J, Berger K, Vicent J, Rivera-Caicedo JP, Wocher M, Verrelst J (2021). Top-of-Atmosphere Retrieval of Multiple Crop Traits Using Variational Heteroscedastic Gaussian Processes within a Hybrid Workflow. Remote Sens.

[26] de Sá NC, Baratchi M, Hauser LT, van Bodegom P (2021). Exploring the Impact of Noise on Hybrid Inversion of PROSAIL RTM on Sentinel-2 Data. Remote Sens.

[27] Rivera-Caicedo JP, Verrelst J, Muñoz-Marí J, Camps-Valls G, Moreno J (2017). Hyperspectral dimensionality reduction for biophysical variable statistical retrieval. ISPRS J Photogramm Remote Sens.

[28] Rasti B, Scheunders P, Ghamisi P, Licciardi G, Chanussot J (2018). Noise Reduction in Hyperspectral Imagery: Overview and Application. Remote Sens.

[29] Morales G, Sheppard JW, Logan RD, Shaw JA (2021). Hyperspectral Dimensionality Reduction Based on Inter-Band Redundancy Analysis and Greedy Spectral Selection. Remote Sens.

[30] Pasolli E, Melgani F, Alajlan N, Bazi Y (2012). Active Learning Methods for Biophysical Parameter Estimation. IEEE Trans Geosci Remote Sens.

[31] Verrelst J, Rivera JP, Gitelson A, Delegido J, Moreno J, Camps-Valls G (2016). Spectral band selection for vegetation properties retrieval using Gaussian processes regression. Int J Appl Earth Obs Geoinf.

[32] Verrelst J, Berger K, Rivera-Caicedo JP (2020). Intelligent Sampling for Vegetation Nitrogen Mapping Based on Hybrid Machine Learning Algorithms. IEEE Geosci Remote Sens Lett.

[33] Tuia D, Volpi M, Copa L, Kanevski M, Muñoz-Marí J (2011). A survey of active learning algorithms for supervised remote sensingimage classification. IEEE J Sel Top Signal Process.

[34] Berger K, Rivera Caicedo JP, Martino L, Wocher M, Hank T, Verrelst J (2021). A Survey of Active Learning for Quantifying Vegetation Traits from Terrestrial Earth Observation Data. Remote Sens.

[35] Settles B (2009). Active Learning Literature Survey.

[36] Rasmussen CE, Williams CKI (2006). Gaussian Processes for Machine Learning.

[37] Camps-Valls G, Verrelst J, Munoz-Mari J, Laparra V, Mateo-Jimenez F, Gomez-Dans J (2016). A survey on Gaussian processes for earth-observation data analysis: A comprehensive investigation. IEEE Geosci Remote Sens Mag.

[38] Verrelst J, Rivera J, Veroustraete F, Muñoz Marí J, Clevers J, Camps-Valls G, Moreno J (2015). Experimental Sentinel-2 LAI estimation using parametric, non-parametric and physical retrieval methods—A comparison. ISPRS J Photogramm Remote Sens.

[39] Verrelst J, Rivera J, Moreno J, Camps-Valls G (2013). Gaussian processes uncertainty estimates in experimental Sentinel-2 LAI and leaf chlorophyll content retrieval. ISPRS J Photogramm Remote Sens.

[40] Wu X, Kumar V, Ross Quinlan J, Ghosh J, Yang Q, Motoda H, McLachlan GJ, Ng A, Liu B, Yu PS (2008). Top 10 algorithms in data mining. Knowl Inf Syst.

[41] Kohavi R, John GH (1997). Wrappers for feature subset selection. Artif Intell.

[42] Saeys Y, Inza I, Larrañaga P (2007). A review of feature selection techniques in bioinformatics. Bioinformatics.

[43] Xue J, Su B (2017). Significant Remote Sensing Vegetation Indices: A Review of Developments and Applications. J Sens.

[44] Haboudane D, Tremblay N, Miller JR, Vigneault P (2008). Remote Estimation of Crop Chlorophyll Content Using Spectral Indices Derived From Hyperspectral Data. IEEE Trans Geosci Remote Sens.

[45] le Maire G, François C, Soudani K, Berveiller D, Pontailler JY, Bréda N, Genet H, Davi H, Dufrêne E (2008). Calibration and validation of hyperspectral indices for the estimation of broadleaved forest leaf chlorophyll content, leaf mass per area, leaf area index and leaf canopy biomass. Remote Sens Environ.

[46] Clevers JGPW (2014). Land Use and Land Cover Mapping in Europe: Practices & Trends.

[47] Glenn EP, Huete AR, Nagler PL, Nelson SG (2008). Relationship Between Remotely-sensed Vegetation Indices, Canopy Attributes and Plant Physiological Processes: What Vegetation Indices Can and Cannot Tell Us About the Landscape. Sensors.

[48] Atzberger C, Richter K, Vuolo F, Darvishzadeh R, Schlerf M (2011). Why confining to vegetation indices? Exploiting the potential of improved spectral observations using radiative transfer models. Remote Sens Agric Ecosyst Hydrol XIII.

[49] Berger K, Atzberger C, Danner M, Wocher M, Mauser W, Hank T (2018). Model-Based Optimization of Spectral Sampling for the Retrieval of Crop Variables with the PROSAIL Model. Remote Sens.

[50] Jolliffe IT, Cadima J (2016). Principal component analysis: a review and recent developments. Philos Trans R Soc Math Phys Eng Sci.

[51] Tagliabue G, Boschetti M, Bramati G, Candiani G, Colombo R, Nutini F, Pompilio L, Rivera-Caicedo JP, Rossi M, Rossini M (2022). Hybrid retrieval of crop traits from multi-temporal PRISMA hyperspectral imagery. ISPRS J Photogramm Remote Sens.

[52] Candiani G, Tagliabue G, Panigada C, Verrelst J, Picchi V, Rivera Caicedo JP, Boschetti M (2022). Evaluation of Hybrid Models to Estimate Chlorophyll and Nitrogen Content of Maize Crops in the Framework of the Future CHIME Mission. Remote Sens.

[53] Verrelst J, Romijn E, Kooistra L (2012). Mapping Vegetation Density in a Heterogeneous River Floodplain Ecosystem Using Pointable CHRIS/PROBA Data. Remote Sens.

[54] Van der Tol C, Berry J, Campbell P, Rascher U (2014). Models of fluorescence and photosynthesis for interpreting measurements of solar-induced chlorophyll fluorescence. J Geophys Res Biogeosci.

[55] Feret JB, François C, Asner GP, Gitelson AA, Martin RE, Bidel LPR, Ustin SL, le Maire G, Jacquemoud S (2008). PROSPECT-4 and 5: Advances in the leaf optical properties model separating photosynthetic pigments. Remote Sens Environ.

[56] Vilfan N, van der Tol C, Muller O, Rascher U, Verhoef W (2016). Fluspect-B: A model for leaf fluorescence, reflectance and transmittance spectra. Remote Sens Environ.

[57] Berger K, Atzberger C, Danner M, D’Urso G, Mauser W, Vuolo F, Hank T (2018). Evaluation of the PROSAIL model capabilities for future hyperspectral model environments: A review study. Remote Sens.

[58] García-Haro FJ, Campos-Taberner M, Munoz-Mari J, Laparra V, Camacho F, Sanchez-Zapero J, Camps-Valls G (2018). Derivation of global vegetation biophysical parameters from EUMETSAT Polar System. ISPRS J Photogramm Remote Sens.

[59] Verger A, Baret F, Camacho F (2011). Optimal modalities for radiative transfer-neural network estimation of canopy biophysical characteristics: Evaluation over an agricultural area with CHRIS/PROBA observations. Remote Sens Environ.

[60] Bacour C, Baret F, Béal D, Weiss M, Pavageau K (2006). Neural network estimation of LAI, fAPAR, fCover and LAI×Cab, from top of canopy MERIS reflectance data: Principles and validation. Remote Sens Environ.

[61] Pacheco-Labrador J, El-Madany TS, van der Tol C, Martin MP, Gonzalez-Cascon R, Perez-Priego O, Guan J, Moreno G, Carrara A, Reichstein M (2021). senSCOPE: Modeling mixed canopies combining green and brown senesced leaves. Evaluation in a Mediterranean Grassland. Remote Sens Environ.

[62] Verhoef W, van der Tol C, Middleton EM (2018). Hyperspectral radiative transfer modeling to explore the combined retrieval of biophysical parameters and canopy fluorescence from FLEX - Sentinel-3 tandem mission multi-sensor data. Remote Sens Environ.

[63] Yang P, van der Tol C, Yin T, Verhoef W (2020). The SPART model: A soil-plant-atmosphere radiative transfer model for satellite measurements in the solar spectrum. Remote Sens Environ.

[64] Verrelst J, Dethier S, Rivera JP, Munoz-Mari J, Camps-Valls G, Moreno J (2016). Active Learning Methods for Efficient Hybrid Biophysical Variable Retrieval. IEEE Geosci Remote Sens Lett.

[65] Douak F, Melgani F, Benoudjit N (2013). Kernel ridge regression with active learning for wind speed prediction. Appl Energy.

[66] Verrelst J, Alonso L, Camps-Valls G, Delegido J, Moreno J (2012). Retrieval of vegetation biophysical parameters using Gaussian process techniques. IEEE Trans Geosci Remote Sens.

[67] Verrelst J, Alonso L, Rivera Caicedo J, Moreno J, Camps-Valls G (2013). Gaussian Process Retrieval of Chlorophyll Content From Imaging Spectroscopy Data. IEEE J Sel Top Appl Earth Obs Remote Sens.

[68] Camps-Valls G, Sejdinovic D, Runge J, Reichstein M (2019). A Perspective on Gaussian Processes for Earth Observation. Natl Sci Rev.

[69] Morata M, Siegmann B, Morcillo-Pallarés P, Rivera-Caicedo JP, Verrelst J (2021). Emulation of Sun-Induced Fluorescence from Radiance Data Recorded by the HyPlant Airborne Imaging Spectrometer. Remote Sens.

[70] De Peppo M, Taramelli A, Boschetti M, Mantino A, Volpi I, Filipponi F, Tornato A, Valentini E, Ragaglini G (2021). Non-Parametric Statistical Approaches for Leaf Area Index Estimation from Sentinel-2 Data: A Multi-Crop Assessment. Remote Sens.

[71] Süβ A, Danner M, Obster C, Locherer M, Hank T, Richter K, Consortium E (2015). Measuring Leaf Chlorophyll Content with the Konica Minolta SPAD-502Plus. GFZ Data Serv.

[72] Zhu J, Tremblay N, Liang Y (2012). Comparing SPAD and atLEAF values for chlorophyll assessment in crop species. Can J Soil Sci.

[73] Siegmann B, Alonso L, Celesti M, Cogliati S, Colombo R, Damm A, Douglas S, Guanter L, Hanuš J, Kataja K (2019). The High-Performance Airborne Imaging Spectrometer HyPlant—From Raw Images to Top-of-Canopy Reflectance and Fluorescence Products: Introduction of an Automatized Processing Chain. Remote Sens.

[74] Danner M, Berger K, Wocher M, Mauser W, Hank T (2019). Fitted PROSAIL parameterization of leaf inclinations, water content and brown pigment content for winter wheat and maize canopies. Remote Sens.

[75] Wocher M, Berger K, Danner M, Mauser W, Hank T (2018). Physically-based retrieval of canopy equivalent water thickness using hyperspectral data. Remote Sens.

[76] Lichtenthaler HK (1987). Methods in Enzymology.

[77] Danner M, Berger K, Wocher M, Mauser W, Hank T (2017). Retrieval of Biophysical Crop Variables from Multi-Angular Canopy Spectroscopy. Remote Sens.

[78] Fang H, Baret F, Plummer S, Schaepman-Strub G (2019). An Overview of Global Leaf Area Index (LAI): Methods, Products, Validation, and Applications. Rev Geophys.

[79] Jonckheere I, Fleck S, Nackaerts K, Muys B, Coppin P, Weiss M, Baret F (2004). Review of methods for in situ leaf area index determination Part I. Theories, sensors and hemispherical photography. Agric For Meteorol.

[80] Ryu Y, Nilson T, Kobayashi H, Sonnentag O, Law BE, Baldocchi DD (2010). On the correct estimation of effective leaf area index: Does it reveal information on clumping effects?. Agric For Meteorol.

[81] Leblanc SG, Chen JM, Fernandes R, Deering DW, Conley A (2005). Methodology comparison for canopy structure parameters extraction from digital hemispherical photography in boreal forests. Agric For Meteorol.

[82] Busetto L, Ranghetti L Prismaread: A Tool for Facilitating Access and Analysis of PRISMA L1/L2 Hyperspectral Imagery v1.0.0. https://irea-cnr-mi.github.io/prismaread/.

[83] R Core Team (2022). R: A Language and Environment for Statistical Computing.

[84] Wutzler T, Migliavacca M, Julitta T (2016). R Package Version 0.5.227.

[85] Pipia L, Amin E, Belda S, Salinero-Delgado M, Verrelst J (2021). Green LAI Mapping and Cloud Gap-Filling Using Gaussian Process Regression in Google Earth Engine. Remote Sens.

[86] Binh NA, Hauser LT, Viet Hoa P, Thi Phuong Thao G, An NN, Nhut HS, Phuong TA, Verrelst J (2022). Quantifying mangrove leaf area index from Sentinel-2 imagery using hybrid models and active learning. Int J Remote Sens.

[87] Marshall M, Belgiu M, Boschetti M, Pepe M, Stein A, Nelson A (2022). Field-level crop yield estimation with PRISMA and Sentinel-2. ISPRS J Photogramm Remote Sens.

[88] Liang L, Geng D, Yan J, Qiu S, Di L, Wang S, Xu L, Wang L, Kang J, Li L (2020). Estimating Crop LAI Using Spectral Feature Extraction and the Hybrid Inversion Method. Remote Sens.

[89] Verrelst J, Rivera JP, Mardashova M, Moreno J (2015). ARTMO’s Global Sensitivity Analysis (GSA) toolbox to quantify driving variables of leaf and canopy radiative transfer models. EARSeL eProc Speical.

[90] Verrelst J, Rivera J, Tol C, Magnani F, Mohammed G, Moreno J (2015). Global sensitivity analysis of the SCOPE model: What drives simulated canopy-leaving sun-induced fluorescence?. Remote Sens Environ.

[91] Liu L, Song B, Zhang S, Liu X (2017). A Novel Principal Component Analysis Method for the Reconstruction of Leaf Reflectance Spectra and Retrieval of Leaf Biochemical Contents. Remote Sens.

[92] Locherer M, Hank T, Danner M, Mauser W (2015). Retrieval of Seasonal Leaf Area Index from Simulated EnMAP Data through Optimized LUT-Based Inversion of the PROSAIL Model. Remote Sens.

[93] Sothe C, Gonsamo A, Arabian J, Snider J (2022). Large scale mapping of soil organic carbon concentration with 3D machine learning and satellite observations. Geoderma.

[94] Ishibashi H, Hino H (2020). Stopping criterion for active learning based on deterministic generalization bounds. arXiv.

