# Supplementary material for: Prototyping Crop Traits Retrieval Models for CHIME: Dimensionality Reduction Strategies Applied to PRISMA Data
Source: Remote Sens (Basel). Author manuscript; Available in PMC 2022 Aug 24. (PMC7613375; doi:10.3390/rs14102448)
Supplement: Supplementary Material [file EMS152688-supplement-Supplementary_Material.pdf]

Appendix A

Table A1. Statistical results obtained with the optimal number of bands for each variable identified by GPR-BAT and validated against the Grosseto and MNI in situ data sets (and theoretical results for FVC and FAPAR).

Variable	Optimal Number of Bands	RMSE	RRMSE	NRMSE	R^2	Train Time (s)	Test Time (s)
**SLA BR**	130	94.794	43.137	28.177	0.001	184.178	0.015
**LAI BR**	6	0.812	38.554	13.533	0.809	1.458	0.006
**CCC BR**	227	0.667	68.775	20.537	0,721	268.194	0.025
**CWC BR**	2	302.114	72.383	27.129	0.669	0.312	0.001
**FAPAR BR**	65	0.045	5.670	4.589	0.967	219.088	0.103
**FVC BR**	218	0.048	6.305	4.872	0.969	658.799	0.097
